# Chlorinated biphenyls effect on estrogen-related receptor expression, steroid secretion, mitochondria ultrastructure but not on mitochondrial membrane potential in Leydig cells

**DOI:** 10.1007/s00441-017-2596-x

**Published:** 2017-03-18

**Authors:** Agnieszka Milon, Malgorzata Opydo-Chanek, Waclaw Tworzydlo, Jerzy Galas, Laura Pardyak, Alicja Kaminska, Anna Ptak, Malgorzata Kotula-Balak

**Affiliations:** 1 Department of Endocrinology, Institute of Zoology, Jagiellonian University in Kraków, Gronostajowa 9, 30-387 Krakow, Poland; 2 Department of Experimental Hematology, Institute of Zoology, Jagiellonian University in Kraków, Gronostajowa 9, 30-387 Krakow, Poland; 3 Department of Developmental Biology and Morphology of Invertebrates, Institute of Zoology, Jagiellonian University in Kraków, Gronostajowa 9, 30-387 Krakow, Poland; 4 Department of Physiology and Toxicology of Reproduction, Institute of Zoology, Jagiellonian University in Kraków, Gronostajowa 9, 30-387 Krakow, Poland

**Keywords:** Calcium level, Chlorinated biphenyls, Estrogen-related receptors, Leydig cells, Mitochondria

## Abstract

To characterize polychlorinated biphenyls (PCBs) action on Leydig cells, PCBs congeners, low-chlorinated (delor 103; d103) and high-chlorinated ones (delor 106; d106) were selected. The cells were treated according to PCBs dose (d103 or d106 0.2 ng/ml in low doses:, or 2 ng/ml in high doses) and type (d103 + d106 in low doses or 103 + 106 in high doses). After 24 h treatment with PCBs, a distinct increase in estrogen-related receptors (ERRs type α, β and γ) expression was revealed. However, the dose- and type-dependent PCBs effect was mostly exerted on ERRα expression. A similar increase in ERRs expression was demonstrated by estradiol but not testosterone, which was without an effect on ERRs. PCBs caused no decrease in the membrane potential status of Leydig cells (either in dose or type schedule) but had severe effects on the mitochondria number and structure. Moreover, PCBs markedly increased calcium (Ca2+) concentration and sex steroid secretion (both androgens and estrogens were elevated). These findings suggest a similar estrogenic action of PCBs congeners (d103 and d106) on Leydig cell function. We report dose- and type-specific effects of PCBs only on Leydig cell ERRs expression. Both delors showed common effects on the mitochondria ultrastructural and functional status. Based on our results, ERRα seems to be the most sensitive to hormonal modulation. The increases in Ca2+ and sex steroid secretion may be due to the activation of ERRs by PCBs binding and/or direct effect of PCBs on ERRs mRNA/protein expression. Nevertheless, to confirm the existence of possible relationships between ERRs signaling (including PCBs as ligands) and mitochondria function in Leydig cells, further intensive studies are needed.

## Introduction

Polychlorinated biphenyls (PCBs) were first manufactured commercially in the late 1920s. In the late 1970s, evidence of their toxicity led to the institution of bans in many countries and to their inclusion on the list of compounds in the Stockholm Convention on Persistent Organic Pollutants of 2001 (UNEP 2001). These chemicals have been used in many different products, including electrical equipment, surface coatings, inks, adhesives, flame-retardants and paints. According to the International Programme on Chemical Safety data (IPCS), PCBs are still being released into the environment, as waste that contains PCBs is incinerated or stored in landfills (IPCS 1992). The intrinsic properties of PCBs, such as high environmental persistence, resistance to metabolism in organisms and tendency to accumulate in lipids, have contributed to their ubiquity in environmental media and have induced concern for their toxic effects after prolonged exposure (Beyer and Biziuk 2009).

PCBs are bioaccumulated and biomagnified and therefore their concentrations increase from one trophic level to the next within the food chain. Aquatic and terrestrial organisms mainly accumulate PCBs. Humans and wildlife consuming contaminated organisms can also concentrate PCBs in their tissues. Thus, PCBs may affect not only individual organisms but ultimately the whole ecosystem (Wang et al. 2016). For instance, in Sweden, the consumption of fatty fish (salmon and herring) from the Swedish east coast Baltic Sea represents a major exposure source of PCBs recently being associated with sperm chromatin integrity defects in Swedish men (Rignell-Hydbom et al. 2005). PCBs are regularly detected in human breast milk, serum and tissues. In addition, these compounds are able to pass through the human placenta (Koppe et al. 1992). Other studies on rats have shown activation of microsomal enzymes of liver by PCBs (especially delor 106) indicating active mechanisms of PCBs metabolism in tissues (Butschak et al. 1978).

For normal male reproductive development and function in both animals and humans, a proper balance between androgens and estrogens is fundamental. Any change in the ratio of sex steroids may disturb the development, maturity and activation status of the male reproductive system and may translate into a wide range of urogenital disorders, affecting fertility (Li et al. 2015). Testicular Leydig cells are responsible for the biosynthesis and secretion of androgens and estrogens. These hormones, in turn, act on Leydig cells in an autocrine manner. Thus, sex hormones regulate endocrine testis function and, additionally, via paracrine signaling, control male reproductive physiology (Hess et al. 1997).

As has been confirmed by multiple studies, PCBs are able to cause hormonal perturbations mainly in developing organisms and have been associated with urogenital maldevelopment in animal models (Cook et al. 2011; Toppari 2008). In addition, chlorinated congener groups of PCBs with more pronounced sex steroid effects are the most relevant for disorders, which in human comprise testicular dysgenesis syndrome (Skakkebaek et al. 2001).

Early observations of PCBs effects were performed in exposed field workers and, together with results of experimental research, principally contributed to understanding the mechanisms of PCBs action on male reproductive function, indicating both estrogen-like and anti-androgenic properties of these chemicals (McLachlan and Arnold 1996; Sikka and Wang 2008). In exposed workers, loss of germ cells (oligo- or azoospermia) was revealed but functioning of Leydig cells and Sertoli cells was unaffected (Slutsky et al. 1999). In addition, clinical studies showed that PCBs interfered with meiosis as workers frequently generated aneuploid spermatozoa (Whorton et al. 1979). Data obtained from laboratory animals emphasized the pleiotropic nature of PCBs effects and the susceptibility of both developing and adult reproductive systems. In sheep exposed *in utero*, PCBs exerted subtle effects on fetal testis proteome but did not significantly disturb testis morphology and testosterone synthesis (Krogenæs et al. 2014). Neonatal PCBs treatment increased testis weight and daily sperm production in adult rats, through induction of hypothyroidism, which led to an increase in Sertoli cell number (Cooke et al. 1996). Other studies showed that exposure to dibromochloropropane impaired the function of both Sertoli and Leydig cells including loss of the latter (Amann and Berndtson 1986; Bjorge et al. 1995; Sod-Moriah et al. 1998; Yoshida et al. 1998). In rats after *in utero* or lactational exposure, PCBs 126 and 169 inhibited conversion of round spermatids between stages VII and VIII. On the other hand, PCBs accelerated virtual maturity of rat Leydig cells by the 15th week, as an increased level of testosterone was found (Yamamoto et al. 2005).

Current data strongly point to PCBs induction of liver, lung, bladder, breast and prostate cancer expansion in rodents and humans (Di Lorenzo et al. 2015; Hashmi et al. 2016; Mutlu et al. 2016; Parada et al. 2016; Pi et al. 2016). Other accumulating epidemiological evidence of elevated tumor risk and mortality in individuals exposed to PCBs led to their recent classification as a human carcinogen by the International Agency for Research on Cancer (IARC 2015). To date, the mechanisms by which PCBs initiate tumors and their development and progression are still unclear. PCBs are able to increase cell oxidative stress, including lipid peroxidation (Gadalla and Andreotti 2015). Also, induction of the cytochrome P450 2B family enzymes has been suggested (Stamou et al. 2015). Moreover, a possible association between leukocyte telomere length and PCBs blood levels in the civilian US adult population has been recently revealed in research on different types of tumors using data from the National Health and Nutrition Examination Survey (Easley et al. 2016; Zhang et al. 2016). In the endometrial adenocarcinoma Ishikawa cells, PCBs affected the expression of inflammatory factors through estrogen receptors (ERs) and the aryl hydrocarbon receptor (AhR), with no adverse effects on estrogen metabolism (Chen et al. 2015). In the rodent male reproductive system, exposure to PCBs decreased serum testosterone and changed the function of the lutropin receptor and activity of both steroidogenic and antioxidant enzymes (Murugesan et al. 2009). In testes of mice treated with PCBs, the estradiol level was decreased, while expressions of ERβ and ERα were increased (Cai et al. 2005).

The above data clearly show the existence of a link between estrogen signaling via ERs and PCBs action in Leydig cells. In our previous study, for the first time, we reported the expression of estrogen-related receptors (ERRs; types α, β and γ) mRNA and protein in mouse Leydig cells (Pardyak et al. 2016). These receptors show a high degree of DNA sequence homology to ERs and the possibility of an overlap, as ERRs can bind to functional estrogen response elements in ER target genes (Huppunen and Aarnisalo 2004). ERRs influence estrogen signaling by either synergizing and/or competing with ERs in the regulation of multiple shared transcriptional targets through nongenomic signaling. Evidence suggests that these receptors are regulated by hormonally active chemicals (Giguère 2002; Liu et al. 2003; Roshan-Moniri et al. 2014; Vanacker et al. 1999).

In recent years, ERRs have been gradually thought to be relevant to reproductive endocrine tumor diseases and even non-reproductive ones (Xu et al. 2016). Based on our results, the expression of ERRs of all types was always higher in tumor cells in comparison to normal ones (Pardyak et al. 2016). In breast cancer, ERRα regulates a number of target genes directing cell proliferation and growth, independently of estrogen receptor alpha (ERα). The pro-angiogenesis factor, vascular endothelial growth factor, expression has been shown to be regulated by ERRα. Knockdown of ERRα in a number of cancer tissues and cell lines significantly reduced tumor growth and malignancy (Ranhotra 2015).

Accumulating evidence indicates that mitochondria are crucial targets for estrogen action and are also a specific estrogen reservoir in the cell (for review, see Liao et al. 2015). In fact, mitochondrial distribution of ERβ has been investigated in various tissue and cell types, including pathological ones. ERs can serve as a transcription factor for mitochondrial DNA genes, e.g., encoding mitochondrial respiratory chain proteins (Leigh-Brown et al. 2010). In estrogen-sensitive tissues, many alterations in mitochondrial function have been demonstrated during estrogen-driven tumorigenesis, including increased mitochondrial respiratory chain protein expression and activity, the induction of mitochondrial gene transcription and the reduction of mitochondrial reactive oxygen species production (Chen et al. 2008).

Considering the above facts, this study was undertaken to better understand and characterize the molecular mechanisms of PCBs action in Leydig cells. Special emphasis will be put on the engagement of ERRs networks as well as the examination of the mitochondria ultrastructure and function in relation to PCBs treatment.

## Materials and methods

### Chemicals

Delor 103 (d103) and delor 106 (d106) (Metrological Institute, Ostrava, Czech Republic; a generous gift to Prof. E. L Gregoraszczuk, Department of Physiology and Toxicology of Reproduction, Institute of Zoology, Jagiellonian University) mixtures were prepared from standard solutions by dilution with ethanol, followed by being aliquoted and refrigerated. Based on dose–response experiments (Leydig cells treated with doses 0.02, 0.2, 2, 20 ng/ml of each compound) (not shown) and studies by Gregoraszczuk et al. (2005) and Grabic et al. (2006), doses of 0.2 and 2 ng/ml of d103 and d106 were selected. A preliminary study showed that 0.2 and 2 ng/ml of delors affected neither Leydig cells viability nor morphology (not shown) but induced detectable changes in mRNA and protein expressions for functional markers of Leydig cells (e.g., lutropin receptor, 3β-hydroxysteroid dehydrogenase, insulin-like 3 peptide) (not shown). Control cells were treated with ethanol. Testosterone (1 μM; Sigma-Aldrich, St Louis, MO, USA) and 17β-estradiol (10 μM; Sigma-Aldrich) were freshly prepared in ethanol and used for comparisons.

The final concentration of ethanol in the medium was less than 0.1%. At this concentration, ethanol had no effect on cell morphology, proliferation or viability (trypan blue exclusion was greater than 95%), (not shown).

The experiments were set up in 24-well plates (Techno Plastic Products, Switzerland) (for real-time RT-PCR analysis, calcium, Ca2+, concentration measurement and radioimmunological measurements of sex steroid level) and in 3-mm-diameter Petri dishes (Techno Plastic Products) (for western blotting, transmission electron microscopy and mitochondrial membrane potential measurement).

### Cell culture and treatments

The mouse Leydig cell line MA-10 was a generous gift from Dr. Mario Ascoli (University of Iowa, Iowa City, IA, USA) and was maintained under a standard technique (Ascoli 1981). Middle passages of MA-10 cells were used for the study. The cells were grown in Waymouth’s media (Gibco, Grand Island, NY, USA) supplemented with 12% horse serum and 50 mg/l of gentamicin at 37 °C in 5% CO_2_. Cells were plated overnight at a density of 1 × 10^5^ cells/ml per well.

Twenty-four hours before the experiments, the medium was removed and replaced with a medium without phenol red supplemented with 5% dextran-coated, charcoal-treated FBS (5% DC-FBS) to exclude estrogenic effects caused by the medium. Next, cells were incubated with d103 or d106 alone or together for 24 h.

### RNA isolation and reverse transcription

Total RNA was extracted from control and delor-treated Leydig cells using TRIzol reagent (Life Technologies, Gaithersburg, MD, USA) according to the manufacturer’s instructions. In order to remove contaminating DNA and the DNase from RNA preparations, the RNA samples were incubated with reagents from the TURBO DNase-free Kit (Abcam, Cambridge, UK). The yield and quality of the RNA were assessed by measuring the A260:A280 ratio in a NanoDrop ND2000 Spectrophotometer (Thermo Scientific, Wilmington, DE, USA) and by electrophoresis. The purified total RNA was used to generate cDNA. A volume equivalent to 1 μg of total RNA was reverse-transcribed using a High-Capacity cDNA Reverse Transcription Kit (Applied Biosystems, Carlsbad, CA, USA), according to the manufacturer’s protocol. cDNA was prepared in a 20-μl volume with the use of the random primers, dNTP mix, RNAse inhibitor and reverse transcriptase (RT). A negative RT reaction (RT enzyme was replaced by water) was performed to detect residual DNA contamination. The RT+ and RT− samples were then subjected to PCR amplification performed in a Veriti Thermal Cycler (Applied Biosystems) with a temperature-cycling program of 10 min at 25 °C, 2 h at 37 °C and 5 min at 85 °C, as described previously (Kotula-Balak et al. 2013). Samples were kept at −20 °C until further analysis. Polymerase chain reactions were performed with a reaction mixture containing 1 μl of cDNA, 10 μM forward and reverse primers obtained from the Institute of Biochemistry and Biophysics PAS (Warsaw, Poland), 10 mM dinucleotide triphosphate, 10× PCR buffer and 2 U of DyNAzyme II polymerase (Finnzymes, Espoo, Finland) in a Veriti Thermal Cycler. Mouse-specific primer sets were devised using Primer3 software from the cDNA sequence available in the Ensembl database (http://www.ensembl.org), (Table 1). Three independent experiments were performed. All PCR products were analyzed by electrophoresis on 1% agarose gel with ethidium bromide together with a ready-load 100-bp DNA ladder marker (Promega, Southampton, UK) and followed by fluorescence digitization with the use of a Bio-Rad GelDoc XR system (Bio-Rad Laboratories, Munchen, Germany).

**Table 1:** Sequences of forward and reverse primers

Genes	Primers (5′–3′)	Product size (bp)	Annealing temperature (°C)	Cycles	References
ERRα	5′- GCCTCTACCCAAACCTCTCT-3′ 5′- AGCCAT CCCTCCTTCGCACA-3′	234	60	40	http://www.ensembl.org (ENSMUST00000173308)
ERRβ	5′- GAGCCATCTTTACCGCTGGA-3′ 5′- CAGCTTGTCAACAGGCAGTG -3′	239	60	40	http://www.ensembl.org (ENSMUST00000136464)
ERRγ	5′- CTTGTAATGGGGTTGCCTC-3′ 5′- TATCACCTTCTGCCGACCT-3′	222	62	35	http://www.ensembl.org (ENSMUST00000152927)
GAPDH	5′- TGAACGGGAAGCTCACTGG-3′ 5′- TACAGCAACAGGGTGGTGA-3′	307	55	30	http://www.ensembl.org (ENSMUSG00000057666)

*ERRα* estrogen-related receptor alpha, *ERRβ* estrogen-related rreceptor beta, *ERRγ* estrogen-related receptor gamma, *GAPDH* glyceraldehyde 3-phosphate dehydrogenase

### Real-time quantitative RT-PCR

Real-time RT-PCR analyses were performed with the StepOne Real-time PCR system (Applied Biosystems) with the same cDNA templates as described earlier and the primers listed in Table 1. Detection of amplification products for ERRα, β, γ and GAPDH was performed with 10 ng cDNA, 0.5 μM primers and SYBR Green master mix (Applied Biosystems) in a final volume of 20 μL. Amplifications were performed as follows: 55 °C for 2 min, 94 °C for 10 min, followed by 40 cycles of 30 s at 62 °C and 45 s 72 °C to determine the cycle threshold (Ct) for the quantitative measurement described previously (Kotula-Balak et al. 2013). To confirm amplification specificity, the PCR products from each primer pair were subjected to melting curve analysis and subsequent agarose gel electrophoresis. The ERRα, β and γ mRNA expressions were normalized to the expression of GAPDH mRNA, with the adjusted expressions in the control group as references (relative quantification, RQ = 1) with the use of the 2 − ΔCt method, as previously described by Livak and Schmittgen (2001).

### Western blotting

For quantification of the ERRs (α, β and γ), protein expressions Leydig cells (control and delor-treated) were cultured in Petri dishes (2 × 10^5^ cells per dish). Having reached ∼80% confluence, the cells were washed twice with ice-cold saline and then the proteins were extracted in 50 μl of radioimmunoprecipitation assay buffer (RIPA; Thermo Scientific, Rockford, IL, USA) and protease inhibitor cocktail (Sigma Chemical, St. Louis, MO, USA). The concentration of the proteins was determined with Bradford reagent (Bio-Rad Protein Assay; Bio-Rad Laboratories GmbH, Munchen, Germany), using bovine serum albumin as a standard. Aliquots (50 μg protein) of cell lysates were used for electrophoresis on 12% mini gel by standard SDS-PAGE procedures and electrotransferred to polyvinylidene difluoride (PVDF) membranes (Millipore Corporate, MA, USA) by a semi-dry transfer cell (Bio-Rad). Then, blots were blocked with 5% nonfat dry milk in TBS, 0.1% Tween 20, overnight at 4 °C with shaking, followed by an incubation with antibodies listed in Table 2. The membranes were washed and incubated with a secondary antibody conjugated with the horseradish-peroxidase labeled goat anti-mouse IgG (Vector Labs, Burlingame, CA, USA) at a dilution 1:1000, for 1 h at RT. Immunoreactive proteins were detected by chemiluminescence with Western Blotting Luminol Reagent (Santa Cruz Biotechnology) and images were captured with a ChemiDoc XRS + System (Bio-Rad Laboratories). All immunoblots were stripped with stripping buffer containing 62.5 mM Tris–HCl, 100 mM 2-mercaptoethanol and 2% SDS (pH 6.7) at 50 °C for 30 min and incubated in a rabbit polyclonal antibody against β-actin (Table 2). Each data point was normalized against its corresponding β-actin data point.

**Table 2:** Primary antibodies used for Western blotting

Antibody	Host species	Vendor	Dilution
ERRα	Rabbit	Abcam cat.no. ab16363	1:1000
ERRβ	Rabbit	Abcam cat.no. ab19331	1:1000
ERRγ	Rabbit	Abcam cat.no. ab49129	1:1000
β-actin	Mouse	Sigma–Aldrich cat. no. A2228	1:3000

*Abbreviations*: *ERRα* estrogen related receptor alpha, *ERRβ* estrogen related rreceptor beta, *ERRγ* estrogen related receptor gamma

To obtain quantitative results, immunoblots were scanned with Image Lab 2.0 (Bio-Rad Laboratories). Then, a bound antibody was revealed using DAB as the substrate. Finally, the membranes were dried and then scanned using an Epson Perfection Photo Scanner (Epson Corporation, CA, USA). Molecular masses were estimated by reference to standard proteins (Prestained SDS-PAGE Standards, Bio-Rad Labs). Quantitative analysis was performed for three separately repeated experiments using a public domain ImageJ software (National Institutes of Health, Bethesda, MD, USA) as described elsewhere (Smolen 1990). The relative protein levels were expressed as arbitrary units.

### TMRE-mitochondrial membrane potential assay

Lipophilic cationic dye, tetramethylrhodamine ethyl ester perchlorate (TMRE; Sigma Aldrich), was used to analyze mitochondrial membrane potential (MMP) in MA-10 cells. A TMRE stock solution was prepared at a concentration of 10 mM in DMSO and stored at −20 °C. Control and delor-treated for 24 h, Leydig cells were incubated with Waymouth’s medium containing 200 nM of TMRE for 30 min at 37 °C. Following incubation, TMRE medium was removed and the cells were washed with phosphate-buffered saline (PBS). Then, cells were detached with trypsin (Sigma-Aldrich), centrifuged and resuspended in a total volume of 0.5 ml PBS. TMRE fluorescence was immediately measured using FACS Calibur flow cytometer (Becton Dickinson). Totals of 10 000 cells were examined per one sample. Data from measurement of Leydig cell samples (after treatment with d103 and d106 in various doses and combinations) were compared with the control using WinMDI 2.8 software and calculated as percent of cells with high mitochondrial potential. In addition, TMRE fluorescence was visualized using an AXIO Scope.A1 fluorescence microscope (Carl Zeiss, Germany).

### Transmission electron microscope analysis

Control and delor-treated Leydig cells were fixed in a mixture of 2% formaldehyde and 2.5% glutaraldehyde in 0.1 M phosphate buffer (pH 7.3) for several days. The suspension was centrifuged, rinsed in the buffer and post-fixed in 2% osmium tetroxide (Sigma-Aldrich) and 0.8% potassium ferrocyanide (Chempur, Poland) in the same buffer for 30 min at 4 °C. After centrifugation (150 *g*, 4 °C) the material was dehydrated in a series of ethanol and acetone (each time followed by centrifugation). Finally, the material was embedded in an epoxy resin (glycid ether 100, formerly known as Epon 812; Serva, Heidelberg, Germany). Ultrathin sections (80 nm thick) were contrasted with uranyl acetate and lead citrate according to standard protocols and analyzed with a Jeol JEM 2100 transmission electron microscope (TEM) at 80 kV (JEOL, Tokyo, Japan) available at the Department of Cell Biology and Imaging, Transmission Electron Microscopy Laboratory, Institute of Zoology, Jagiellonian University, Krakow.

### Sex steroid concentration measurement

Control and delor-treated Leydig cell culture media (100 μl) were collected for determination of sex steroid content (testosterone and estradiol, respectively) using the radioimmunological technique described elsewhere (Dufau et al. 1972; Hotchkiss et al. 1971; Kotula-Balak et al. 2011, 2013; Starowicz et al. 2015). Testosterone levels were assessed using [1,2,6,7-^3^H]-testosterone (specific activity 110 Ci/mmol; American Radiolabeled Chemicals) as a tracer and rabbit antibody against testosterone-3-0-CMO:BSA (a gift from Dr. B. Ričařova, Institute of Radiology, Czech Academy of Sciences, Prague, Czech Republic). The lower limit of sensitivity was 5 pg. Cross-reaction of this antibody was 18.3% with dihydrotestosterone, 0.1% with androstenedione and less than 0.1% with other major testis steroids. Coefficients of variation within and between assays were below 5.0 and 9.7%, respectively.

Estradiol concentrations were measured using [2,4,6,7-^3^H]-estradiol (specific activity 81 Ci/mmol: American Radiolabeled Chemicals) as a tracer and rabbit antibody against estradiol-17-O-carboxymethyloxime: BSA (a gift from Prof. R. Rembiesa, Institute of Pharmacology, Polish Academy of Sciences, Krakow, Poland). The lower limit of sensitivity of the assays was 5 pg. Cross-reaction was 1% with keto-oestradiol-17b, 0.8% with oestrone, 0.8% with oestriol, 0.01% with testosterone and less than 0.1% with major ovarian steroids. Coefficients of variation within and between assays were below 4 and 7.5%, respectively. Assays were validated by demonstrating parallelism between serial dilutions of culture media and standard curve. Coefficients of variation within and between each assay were 7.6 and 9.8%, respectively. The recovery of unlabeled steroids was also assessed (never less than 90%). In addition to monitoring intra-assays and inter-assays, assay quality control was assessed by control samples representing low, medium and high concentrations of measured hormones. Samples were counted in a scintillation counter (LKB 1209 RACKBETA; LKB, Turku, Finland). The concentrations of sex steroids were calculated as pg/10^5^ cells.

### Determination of Ca2+ levels

Control and delor-treated Leydig cells were lysed in cold RIPA buffer (Thermo Scientific) and then sonicated for 60 s on ice and centrifuged at 10,000 *g* for 15 min. Ca2+ was estimated in the supernatants using Arsenazo III (Sigma–Aldrich) according to the modified method of Michaylo and Ilkova (1971). The intensity of the purple complex formed with the reagent was read at 600 nm in a spectrophotometer (Labtech LT-4000MS; Labtech International, Uckfield, UK) with Manta PC analysis software. The proteins were estimated by modified Lowry’s method (Lowry et al. 1951). Concentrations of Ca2+level in samples of Leydig cells after treatment with d103 and d106 in various doses and combinations were compared with the control, which was arbitrarily set at 1. The Ca2+ levels were calculated as μg/ml.

### Statistics

Three biological repeats of each sample (*n* = 3) and three independent experiments were performed. Each variable was tested using the Shapiro–Wilks *W* test for normality. The homogeneity of variance was assessed with Levene’s test. Comparisons were performed by one-way ANOVA, followed by Dunnett’s post hoc test (GB-STAT software, v.7.0; Dynamic Microsystems) to determine the significant differences between mRNA expression levels and Ca2+ level and sex hormone levels. Statistical analyses were performed on raw data using Statistica 10 software (StatSoft, Tulsa, OK, USA). Data were presented as means ± SD. Data were considered statistically significant at *P < 0.05, **P < 0.01, ***P < 0.001.

## Results

### Expression of ERRα, ERRβ and ERRγ mRNA in Leydig cells after polychlorinated biphenyles treatment

**Fig. 1:**
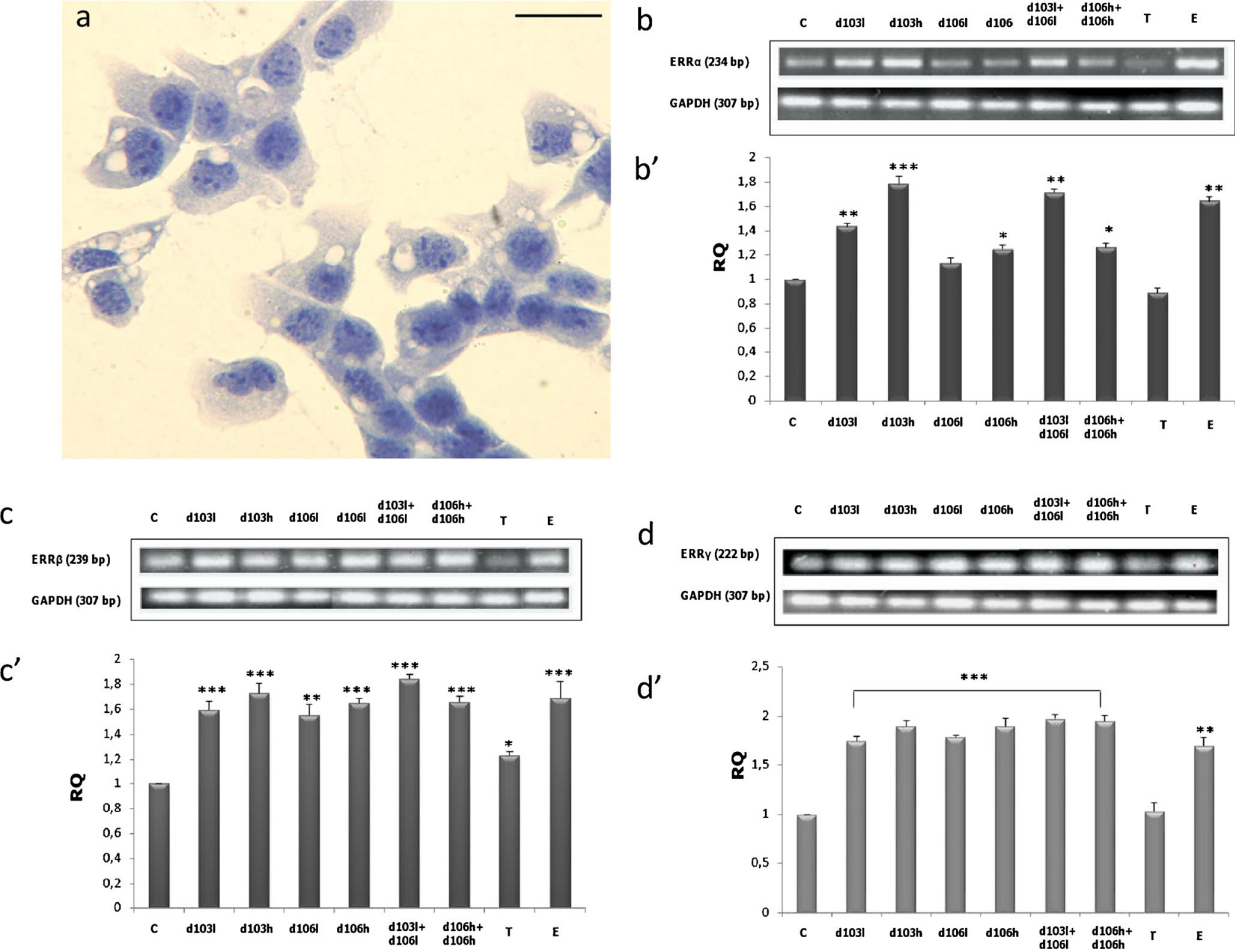
Effects of delor 103 (*d103*) and delor 106 (*d106*) alone and in dose and type combinations on ERRα, ERRβ and ERRγ mRNA expression in Leydig cells. **a** Representative microphotograph of MA-10 cells culture counterstained with hematoxylin. *Bar* 20 μm. **b**–**d** Representative gel electrophoresis of qualitative expression: ERRα (**b**), ERRβ (**c**) and ERRγ (**d**). **b’**–**d’** Relative expression (relative quantification; RQ) of mRNA for ERRα (**b’**), ERRβ (**c’**) and ERRγ (**d’**) determined using real-time RT-PCR analysis 2 − ΔCt method. As an intrinsic control, the GAPDH mRNA level was measured in the samples. Each sample of cellular mRNA was measured in three repeats. RQ from three separate analyses is expressed as means ± SD. *Asterisks* show significant differences between expression of ERRs mRNA in Leydig cells exposed to low (0.2 ng/ml) and high (2 ng/ml) doses of d103 and d106 alone and in combinations (d103l + d106l and d103h + d106h), respectively for 24 h. Testosterone (*T*; 10 μM) and 17β-estradiol (*E2*; 1 μM) were used for comparison of d103 and d106 action. *P < 0.05, **P < 0.01 , ***P < 0.001

To determine whether PCBs alter the expression of ERRα, ERRβ and ERRγ at the mRNA level, MA-10 Leydig cells (Fig. 1a) were treated with low (0.2 ng/ml) and high (2 ng/ml) doses of delor 103 (d103l and d103h, respectively) and delor 106 (d106l and d106h, respectively) alone or in combinations (d103l + d106l and d106h + d106h, respectively) and were analyzed by RT-PCR (Fig. 1b–d, b’–d’). To examine d103 and d106 action in Leydig cells, concomitant treatment of cells with testosterone (1 μM) and 17β-estradiol (10 μM) was performed.

Real-time RT-PCR analysis was applied to quantitatively evaluate ERRs mRNA expression in Leydig cells that was normalized to GAPDH expression (Fig. 1b–d). Relative levels of the transcripts in samples of Leydig cells after treatment with d103 and d106 in various doses and combinations were compared with the control, which was arbitrarily set at 1.

The analysis revealed ERRs transcripts in respective lengths corresponding to *ERRα* (234 bp), *ERRβ* (229 bp) and *ERRγ* (222 bp), respectively (Fig. 1b–d, b’–d’). Independently of PCBs dose and type, expression of *ERRα*, *ERRβ* and *ERRγ* in Leydig cells markedly increased (*P* < 0.05, *P* < 0.01, *P* < 0.001) (Fig. 1b–d, b’–d’). After treatment with estradiol, an increase (*P* < 0.05) in mRNAs expressions of all ERRs was also revealed. In contrast, testosterone did not cause changes in ERRs mRNA expressions. The most visible dose- and type-dependent effects of PCBs were reflected in changes of *ERRα* expression (Fig. 1b, b’). In detail, high doses of d103 and d106 significantly (*P* < 0.05, *P* < 0.001) increased *ERRα* expression, more than their low doses (*P* < 0.01, *P* < 0.001). In contrast, a mixture of delors in low doses exerted a more pronounced effect than a high dose mixture (Fig. 1b, b’).

Distinct changes in mRNA expression of ERRβ (*P* < 0.01, *P* < 0.001) and ERRγ (*P* < 0.001) after type combinations of PCBs treatment were revealed, while a dose-dependent effect was only subtle (Fig. 1c, d, c’, d’).

### Expression of ERRα, ERRβ and ERRγ protein in Leydig cells after polychlorinated biphenyles treatment

**Fig. 2:**
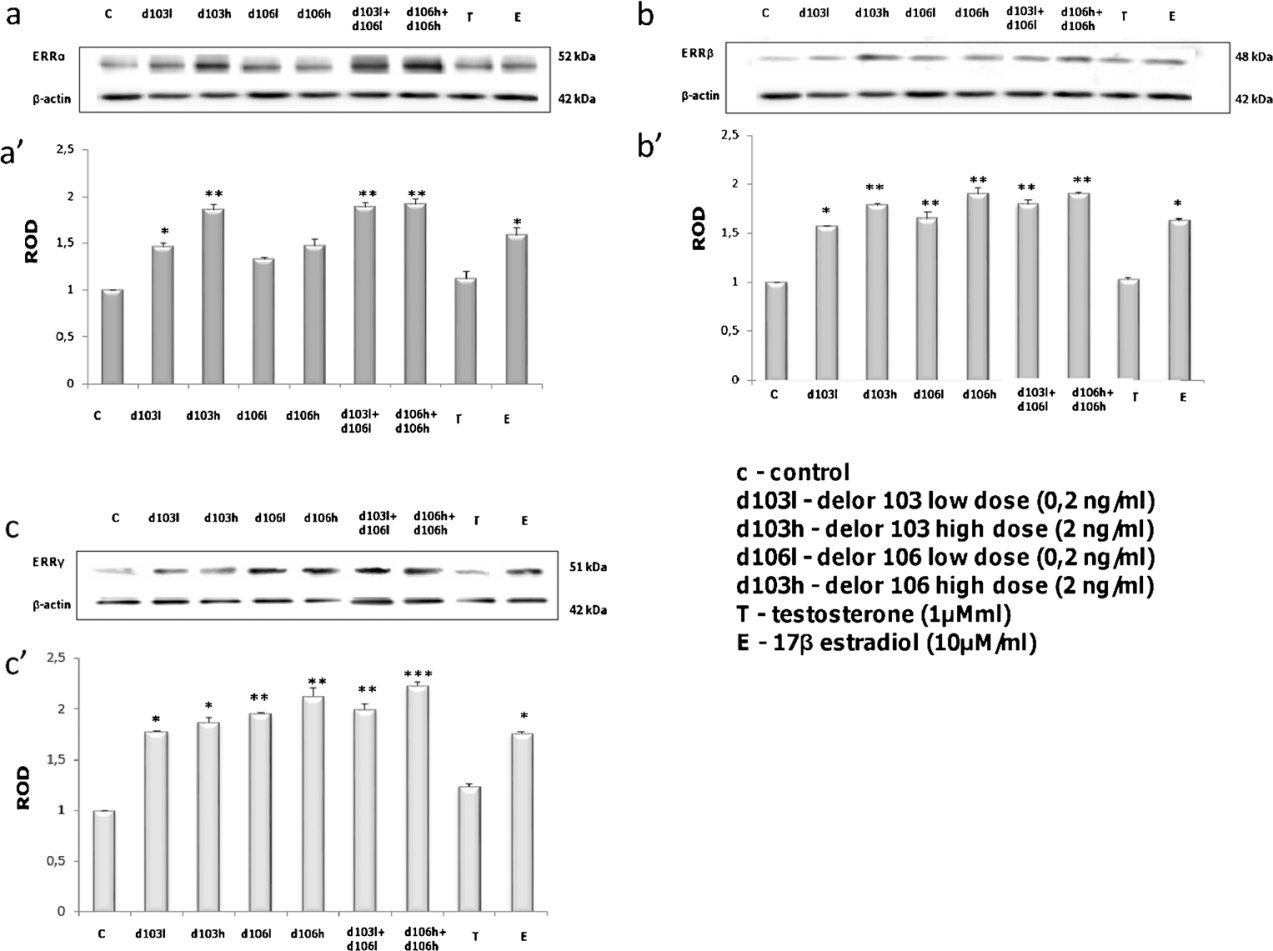
Effects of delor 103 (*d103*) and delor 106 (*d106*) alone and in dose and type combinations on ERRα, ERRβ and ERRγ protein expression in Leydig cells. **a**–**c** Representative blots of qualitative expression: ERRα (**a**), ERRβ (**b**) and ERRγ (**c**). **a’**–**c’** Relative expression of ERRα (**a’**), ERRβ (**b’**) and ERRγ (**c’**) proteins (arbitrary units). The relative amount of respective proteins normalized to β-actin. Each sample of cellular total protein was measured in three repeats. ROD from three separate analyses is expressed as means ± SD. *Asterisks* show significant differences between expression of ERRs protein in Leydig cells exposed to low (0.2 ng/ml) and high (2 ng/ml) doses of d103 and d106 alone and in combinations (d103l + d106l and d103h + d106h), respectively for 24 h. Testosterone (*T*; 10 μM) and 17β-estradiol (*E2*; 1 μM) were used for comparison of d103 and d106 action. *P < 0.05, **P < 0.01 , *P < 0.001

Western blot analyses were performed to confirm that Leydig cells had actively translated the mRNAs ERRα, ERRβ and ERRγ as well as to assess changes in the levels of ERRα, ERRβ and ERRγ protein in control and PCBs-treated cells (d103l and d103h, respectively; d106l and d106h, respectively alone or in combinations: d103l + d106l and d106h + d106h, respectively) as well as cells treated with testosterone and estradiol. Immunodetectable ERRα, ERRβ and ERRγ were observed as single bands near the 52, 48 and 51 kDa position; respectively of the SDS gel in lysates of the control and treated Leydig cell (Fig. 2a–c, a’–c’). After stripping, immunoblots were processed for actin. The bands representing each data point were densitometrically scanned and the data obtained were normalized against its corresponding β-actin. The protein level within the control cells was arbitrarily set as 1, against which statistical significance was analyzed.

An increase in bands intensities for all types of ERRs when compared to control was found (Fig. 2a–c, a’–c’). A distinct dose- and type-dependent increase (*P* < 0.01, *P* < 0.001) in the intensities of the bands was detected especially for ERRα and also for ERRβ proteins (Fig. 2a, b, a’, b’) while this was not clearly revealed for ERRγ (*P* < 0.01, *P* < 0.001), (Fig. 2c, c’). In addition, Leydig cells treated with d103 showed an increase of ERRs expression in comparison to d106 ones (Fig. 2a–c, a’–c’). However, an increase of expression of ERRα was the lowest compared to ERRβ and ERRγ (Fig. 2a, a’). Intensities of bands for all ERR types in cells treated with PCBs, used in combinations of doses and types, were always increased compared to controls and did not show any distinct differences in expressions of all ERRs (Fig. 2a–c, a’–c’). The intensities of the bands for ERRs after estradiol treatment were significantly increased (*P* < 0.05) whereas testosterone treatment caused no effect on ERRs expression (Fig. 2a–c, a’–c’).

### Leydig cell mitochondrial membrane potential after polychlorinated biphenyles treatment

**Fig. 3:**
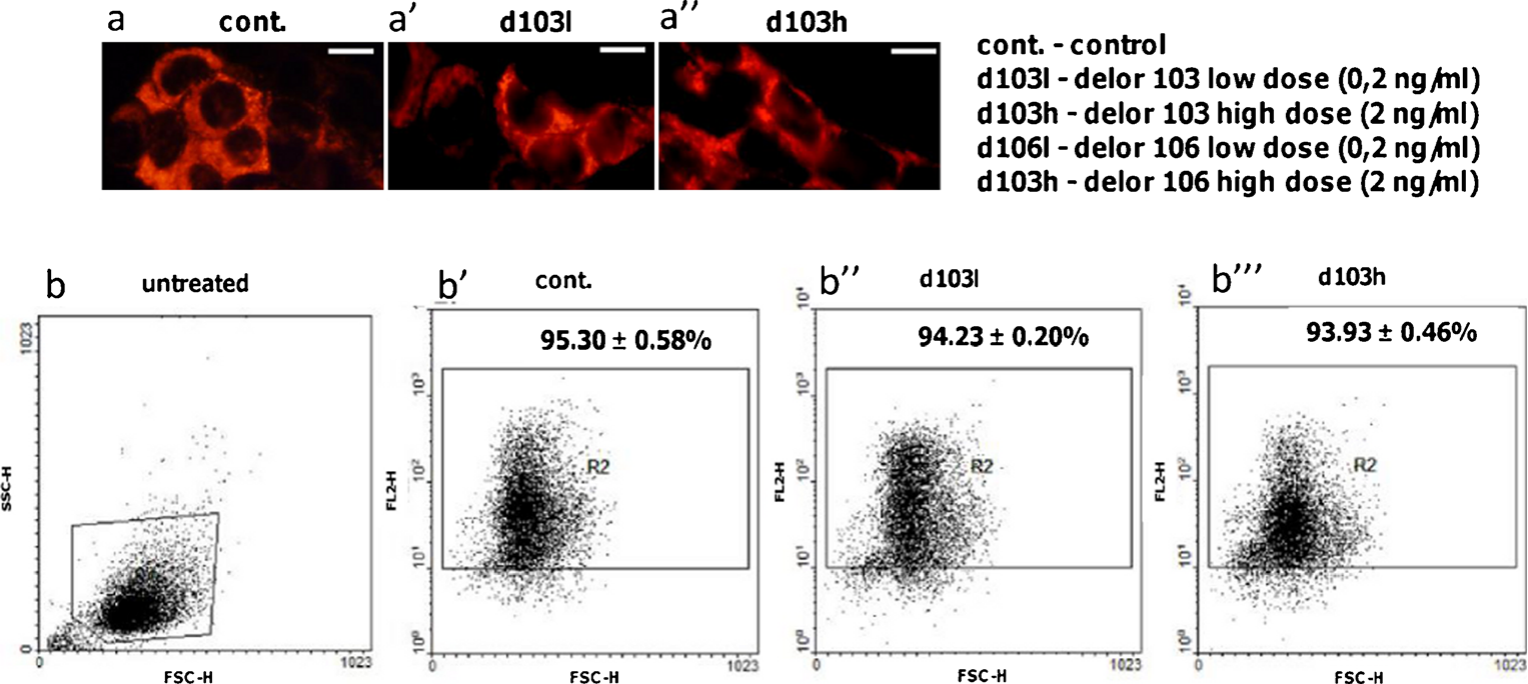
Effects of delor 103 (*d103*) and delor 106 (*d106*) alone and in dose and type combinations (not shown) on mitochondrial membrane potential in Leydig cells. **a**–**a**” Representative microphotographs of control and delors d103h- and d106h (2 ng/ml)-treated Leydig cells stained with tetramethylrhodamine ethyl ester perchlorate. *Bars* 20 μm. **b**–**b”’** Representative FCS/SSC dot plots; **b** untreated Leydig cells; **b’**–**b”’** representative control, d103l and d103h-treated Leydig cells stained with TMRE. Gate *R2* (**b**) indicates cells with high mitochondrial potential. No differences are visible between membrane mitochondrial potential of control cells and those treated with low (0.2 ng/ml) and high (2 ng/ml) doses of d103 and d106 alone (d106l and d106h, not shown) and in combinations (d103l + d106l and d103h + d106h; not shown), respectively for 24 h. Each cellular sample was measured in three repeats. Data from three separate analyses are expressed as means ± SD

Mitochondrial membrane potential in Leydig cells treated with PCBs was examined both qualitatively and semi-quantitatively (representative microphotographs and dot plots from d103l and d103h treatment; Fig. 3a–a”, b–b””; Table 3a). Only a slight decrease in mitochondrial membrane potential was revealed after treatment with different PCBs doses (Fig. 3a–a”, b–b””; Table 3a) and combinations (Table 3a). Both low and high doses and combinations of d103 and d106 (Table 3a) did not lead to a significant decrease of mitochondrial membrane potential. In detail, only d106 in dose and both delor combinations exerted a slightly more potent effect than other treatments. Testosterone and estradiol did not show any diverse action on mitochondrial membrane potential that was still slightly decreased.

**Table 3:** Mitochondrial membrane potential (*a*), sex steroid secretion (*b*) and Ca2+ concentration (*c*) in Leydig cells after polychlorinated biphenyles treatment

(a) Mitochondrial membrane potential (cell % with high membrane potential)		(b) Sex hormones concentration (pg/10^5^ cells)			(c) Ca2+ concentration (μg/ml)	
			Testosterone	Estradiol		
control	95.30 ± 0.58	control	52.05 ± 23.07	544.06 ± 57.01	Control	1
d103l	94.23 ± 0.20	d103 l	121.11 ± 43.01*	854.06 ± 12.09**	d103 l	2.76 ± 0.10*
d103h	93.93 ± 0.46	d103h	129.00 ± 13.01*	892.01 ± 78.14**	d103h	2.90 ± 0.08*
d106l	92.36 ± 1.14	d106l	164.06 ± 48.02*	901.02 ± 68.01**	d106l	2.67 ± 0.08*
d106h	90.29 ± 0.33	d103h	159.05 ± 22.00*	919.00 ± 99.00**	d103h	2.10 ± 0.09*
d103l + d106l	91.15 ± 0.29	d103l + d106l	133.10 ± 57.00*	912.05 ± 15.06**	d103l + d106l	2.87 ± 0.10*
d103h + d106h	87.00 ± 1.00	d103h + d106h	145.09 ± 12.04*	924.04 ± 77.02**	d103h + d106h	2.72 ± 0.05*
T	90.05 ± 1.90				T	2.77 ± 0.08*
E2	89.01 ± 1.60				E2	2.93 ± 0.10*

*Abbreviations*: *c* control, *d103 l* delor 103 low dose (0,2 ng/ml), *d103h* delor 103 high dose (2 ng/ml), *d106l* delor 106 low dose (0,2 ng/ml), *d106l* delor 106 low dose (0,2 ng/ml), *d103h* delor 106 high dose (2 ng/ml), *T* testosterone (1 μM), *E*-*17β* estradiol (10 μM)

*P < 0.01 and **P < 0.001 for (b) and *P < 0.001 for (c) analysis

### Leydig cell mitochondria ultrastructure after polychlorinated biphenyles treatment

**Fig. 4:**
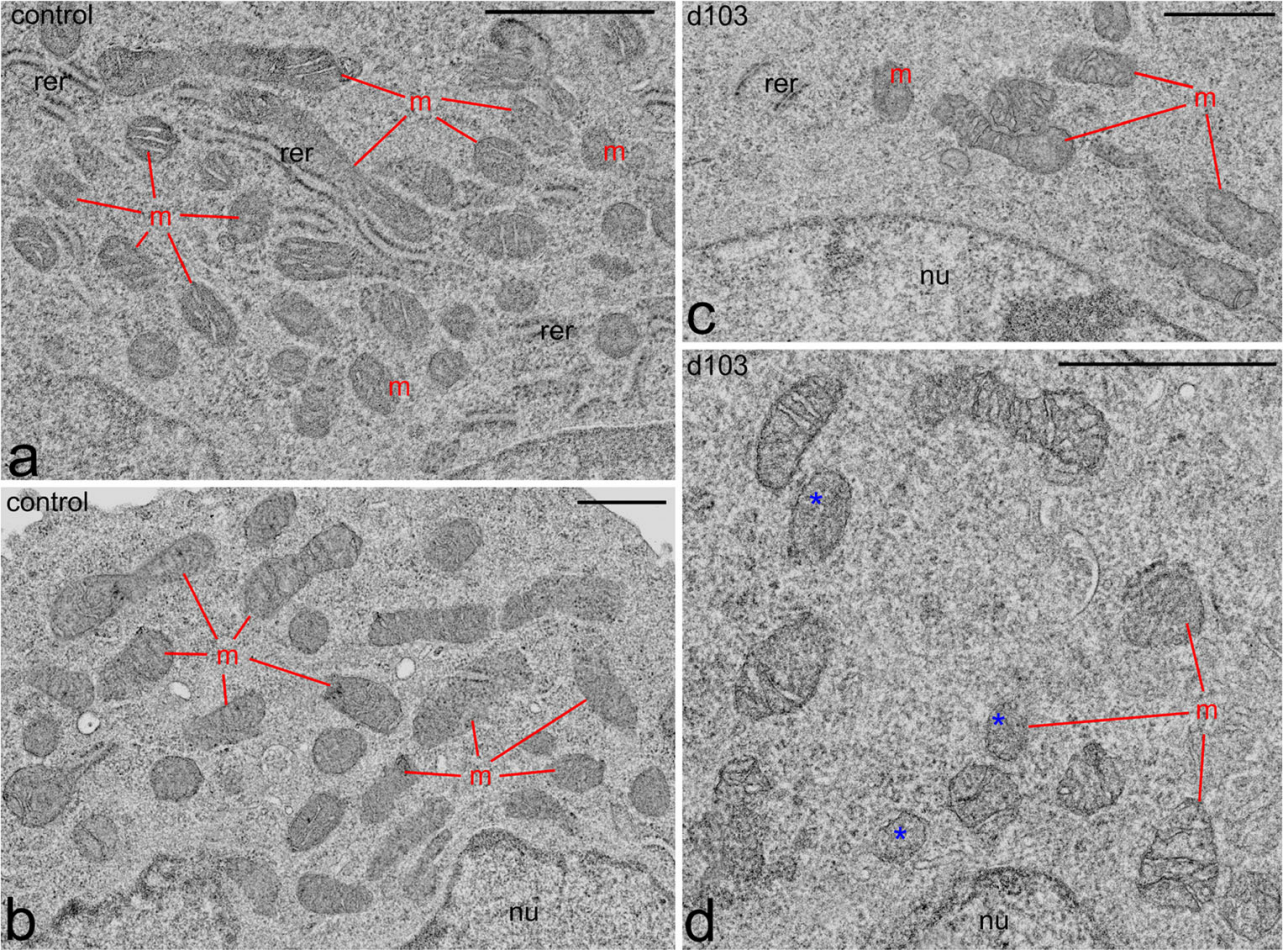
Effects of delor 103 (*d103*) on mitochondria ultrastructure in Leydig cells. From each cellular sample, an epoxy resin block was prepared that was cut into at least three ultrathin sections that were analyzed. Fragment of the perinuclear cytoplasm in control (**a**, **b**) and d103h (2 ng/ml)-treated (**c**, **d**) Leydig cells (representative microphotographs for dose and type schedule used in this study). Note that in the control, Leydig cells mitochondria (*m*) form huge aggregates in close vicinity to the cell nucleus (*nu*). In d103-treated Leydig cells, the mitochondria are less numerous and their morphology is altered. In detail, they are irregularly shaped and swollen (*red arrows*). Note the absence of mitochondrial cristae in some mitochondria (*blue asterisks*). Elements of RER (*rer*). *Bars* 1 μm

Although, both d103 and d106 had a limited effect on mitochondrial membrane potential, analyses of serial ultrathin sections of control and experimental Leydig cells at the level of electron microscopy were performed to check whether even slightly decreased mitochondrial potential was reflected by the morphology of these organelles in PCB-treated cells. Careful analyses showed that, in control cells, in close vicinity to more or less the spherical cell nucleus rather huge accumulations of numerous mitochondria were located (Fig. 4a, b). The mitochondria were variously shaped: some were spherical and rod-shaped, other were markedly elongated. Their morphology all showed signs of their high activity. They had numerous well-developed and clearly visible cristae immersed in a relatively electron-dense matrix (Fig. 4a, b). Numerous cisternae of rough endoplasmic reticulum (coated with ribosomes) were also located in the cytoplasm between mitochondria (Fig. 4a, b).

In delor-treated Leydig cells (both in dose and type schemes), the mitochondria were scarce and were distributed within the cytoplasm rather uniformly and never formed large accumulations (Fig. 4c; representative microphotograph from d103h treatment, for dose and type schedule). The morphology of the mitochondria was clearly altered. They were irregularly shaped and most of them were remarkably distended (Fig. 4c, d; arrows). The mitochondrial cristae were less numerous and not as distinct. At least some of the mitochondria of delor-treated cells had no cristae at all (Fig. 4d; asterisks). Cisternae of smooth (devoid of ribosomes) and rough endoplasmic reticulum were located between the mitochondria (Fig. 4c).

### Sex steroid secretion by Leydig cells after polychlorinated biphenyles treatment

**Fig. 5:**
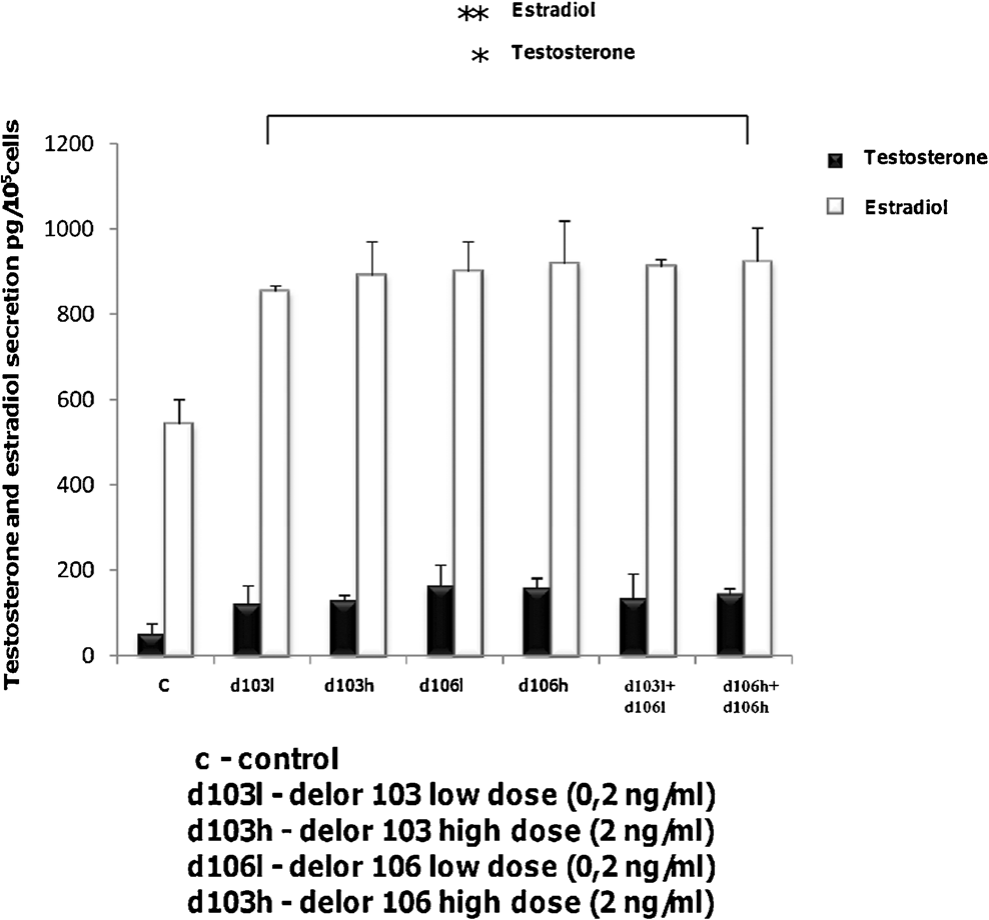
Effects of delor 103 (*d103*) and delor 106 (*d106*) alone and in dose and type combinations on sex steroid secretion in Leydig cells. Each sample of culture medium was measured in three repeats. Data from three separate analyses are expressed as means ± SD. *Asterisks* show significant differences between testosterone and estradiol secretions by Leydig cells exposed to low (0.2 ng/ml) and high (2 ng/ml) doses of d103 and d106 alone and in combinations (d103l + d106l and d103h + d106h), respectively, for 24 h. *P < 0.01, **P < 0.001

Exposure of Leydig cells to PCBs (d103l and d103h, respectively; d106l and d106h, respectively) alone or in combinations (d103l + d106l and d106h + d106h, respectively) markedly elevated secretion of both androgens (*P* < 0.01) and estrogens (*P* < 0.001),(Fig. 5; Table 3b). The increase in sex steroid secretion after PCBs was revealed independently of treatment regime (Fig. 5; Table 3b).

### Ca2+ concentration in Leydig cells after polychlorinated biphenyles treatment

**Fig. 6:**
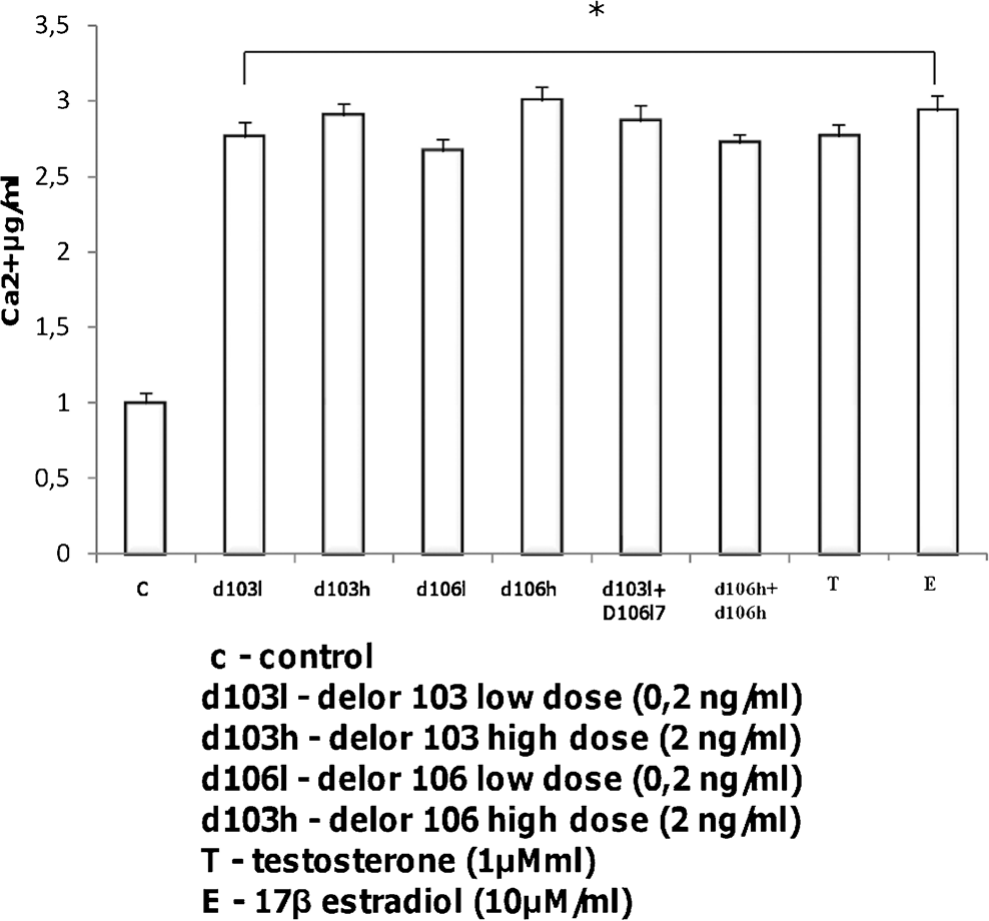
Effects of delor 103 (*d103*) and delor 106 (*d106*) alone and in dose and type combinations on Ca2+ concentration in Leydig cells. Each sample of cellular supernatant was measured in three repeats. Data from three separate analyses are expressed as means ± SD. *Asterisks* show significant differences between Ca2+ concentration in Leydig cells exposed to low (0.2 ng/ml) and high (2 ng/ml) doses of d103 and d106 alone and in combinations (d103l + d106l and d103h + d106h), respectively for 24 h. Testosterone (*T*; 10 μM) and 17β-estradiol (*E2*; 1 μM) were used for comparison of d103 and d106 action. *P < 0.001

Mitochondria accumulate Ca2+ and regulate intracellular Ca2+ levels control sex-steroid biosynthesis. Analysis of Ca2+ concentration in PCBs-treated Leydig cells revealed that, independently of dose and type schedule, PCBs significantly increased (*P* < 0.001) the Ca2+ level in Leydig cells (Fig. 6; Table 3c). High doses of both d103 and d106 increased Ca2+ concentrations slightly more than low doses. In contrast, the combination of low PCBs doses caused a slightly greater increase of the Ca2+ level than the combination of the high ones. Treatment with testosterone and estradiol increased the Ca2+ level like PCBs.

## Discussion

Polychlorinated biphenyles (PCBs) used as dielectric fluid, flame retardants, ink solvents, pesticides and plasticizers are biodegraded more slowly in the environment than are many other organic chemicals. The low water solubility and the low vapor pressure of PCBs, coupled with air, water and sediment transport processes, means that they are readily transported from local or regional sites of contamination to remote areas (Beyer and Biziuk 2009). PCBs still present and accumulated in the environment will have a long-term effect on the environment, wildlife and humans. The results of reproductive toxicity research have indicated that PCBs pose the greatest risk of the chemicals studied (Wang et al. 2011; Wei et al. 2011). Due to growing global, regional and national trends in male infertility, partly attributed to environmental contamination, understanding the target and mechanisms of action of endocrine disrupting chemicals as well as the elaboration of prevention and treatment strategies are constantly needed (Inhorn and Patrizio 2015).

Our study is the first report demonstrating PCBs effect on estrogen-related receptor expression (ERRs), calcium (Ca2+) signaling as well as their mitochondria ultrastructure and function in Leydig cells. It was found that PCBs (delor 103; d103) and (delor 106; d106) alone or in combination acted directly on the expression of ERRs genes and proteins. In line with multiple studies confirming that in Leydig cells various proteins are targets of endocrine disruptors (Svechnikov et al. 2010), this result indicates that PCBs may act via ERRs and/or may affect these receptors in Leydig cells. Modulation of ERRs expression by PCBs reflects the estrogenic properties of these chemicals, while acting on Leydig cells. The synthetic estrogen, diethylstilbestrol, binds all ERRs whereas the estrogen receptor modulator (4-hydroxytamoxifen) was reported to bind ERRβ and ERRγ (Coward et al. 2001; Tremblay et al. 2001). Whether an estrogenic ligand is required for the activation of the ERRs is an unclear and controversial issue that seems to be related to the cell type, cell physiological condition and type of regulatory factors (hormones, proteins or other signaling molecules) (Kamei et al. 2003; Vanacker et al. 1999; Xie et al. 1999; Zhang and Teng 2000). It is worth noting that several natural phytoestrogens (isoflavones: genistein, daidzein and biochanin A; and flavone: 6, 3, 4-trihydroxyflavone) and bisphenol A have also been identified as ERRs ligands with agonistic activities (Roshan-Moniri et al. 2014; Takayanagi et al. 2006). Inclusion of ERRs into the group of receptors for estrogen-mimicking chemicals [nuclear and membrane progesterone, androgen and estrogen receptors, as well as other types: peroxisome proliferator-activated receptor (PPAR) and AhR in Leydig cells; Jeung and Choi 2010; Kotula-Balak et al. 2011, 2013; Rouiller-Fabre et al. 2015; Svechnikov et al. 2010, 2015] needs to be taken into consideration.

In Leydig cells exposed to PCBs, expression of ERRα showed the most prominent increase, which was dependent on the dose and type of used chemicals. As we reported previously, among ERRs, expression of ERRα was always lowest regardless of the cell of origin (primary or tumor) (Pardyak et al. 2016). Thus, ERRα seems to be more sensitive to hormonal treatment than other ERRs, while ERRγ has the lowest sensitivity. Additionally, this result points to the modulation of ERRs function by PCBs and estrogen. The latter compound also markedly increased ERRs mRNA and protein expression in Leydig cells. Recently, the expression of ERRα was found to be upregulated by estrogen in mouse uterus and heart (Liu et al. 2003). Interestingly, expression of this receptor was also upregulated in liver by fasting (Ichida et al. 2002) and in brown fat by exposure to cold (Schreiber et al. 2004).

The potential estrogenic activity of PCBs has been demonstrated in vitro (breast cancer; MCF-7 cells) and in vivo (rat adipose tissue, brain) (Arcaro et al. 1999; Hany et al. 1999; Shekhar et al. 1997). In female reproductive tissues, PCBs exerted an estrogenic effect through repression of the Wnt7a pathway (Ma and Sassoon 2006). These studies revealed that the estrogenic properties of PCBs were weak (Lind et al. 1999), whereas some PCB mixtures in other systems (liver cancer cells, breast cancer cells; MDA-MBA-231, mice uterus) exhibited antiestrogenic activity (Ramamoorthy et al. 1997).

Based on very recent findings, it has been postulated that ERRs occupy a central node at the interface of cancer and metabolism (Tam and Giguère 2016). Therefore, modulation of ERRs activity represents a valuable strategy to induce metabolic vulnerability in tumors of various origins, achieving a more comprehensive response to current therapies.

In the tumor mouse Leydig cells used in this study, PCBs did not modulate mitochondrial function. In rat brain in vitro, PCBs inhibited mitochondrial Ca2+ uptake (Kodavanti and Ward 2005), while in brain and liver cells of rats, significant reductions of oxygen consumption and respiratory chain complexes II and III have been demonstrated (Ounnas et al. 2016). In addition, induction of mitochondria dysfunction by PCBs in SH-SY5Y (neuroblastoma cells) and Vero cells (kidney epithelial cell line) was recently shown by Shen et al. (2011) and Cocco et al. (2015). In the present study, we reported modulation of ERRs expression and function as well as ultrastructure of mitochondria in Leydig cells treated with PCBs. Current studies have shown a strong influence of ERRα on the coordination of mitochondria physiology (mitochondria biogenesis and genome regulation) (Ranhotra 2015). It should also be noted that ERRs control nearly half of the proteins encoded by the mitochondrial genome. Thus, the above data clearly concern ERRs regulation and signaling in mitochondria physiology. Wang et al. (2015) recently confirmed that ERRα and ERRγ are essential, especially for normal mitochondria functions, by coupling cellular energy metabolism with energy consumption processes in cardiac cells. In addition, these authors reported the regulation of Ca2+ homeostasis in cardiomyocytes by ERRs, as was observed in skeletal muscle cells by Rangwala et al. (2010). Further evidence has suggested that PPARγ coactivator-1α and ERRα work in concert to regulate mitochondrial biogenesis (Schreiber et al. 2004) and the oxidative phosphorylation program (Mootha et al. 2004), by directly influencing the expression of controlling genes. Also, ERRα is an important mediator of adaptive mitochondrial biogenesis under conditions of increased physiological stress, as evidenced by the inability of ERRα knock-out mice to regulate body temperature upon cold challenge (Villena et al. 2007).

Contrary to the undisturbed mitochondria function in PCBs-treated Leydig cells, altered mitochondria ultrastructure and the decreased number of these organelles are signs of degeneration, which can be interpreted as the initial stages of the mitophagy, i.e., the selective degradation of defective mitochondria by autophagy (Ding and Yin 2012). On the other hand, Gilroy et al. (1998) reported an increased volume of mitochondria in rat hepatocytes after PCBs treatment. Two types of abnormal mitochondria, in PCBs-treated rat hepatocytes, named Type I and Type II, were defined by Peng et al. (1997): the former comprised mitochondria that had cristae lying parallel to the long axis of the organelle and the latter showed C- or ring-shaped profiles. Data analysis revealed a trend toward an increase in abnormal mitochondria volume in the cells as the congener concentration was elevated. Also, in another study, mitochondrial abnormalities such as dumbbell shapes and cristae, which were oriented parallel to the long axis of the mitochondria, were presented (MacLellan et al. 1994). Toxicity of PCB 1232 on mitochondria of fish *Arius caelatus* (*Valenciennes*) has also been well documented (Selvarani and Rajamanickam 2003).

Biosynthesis of steroids in Leydig cells is a multistep process that is based on the physical association between mitochondria and smooth endoplasmic reticulum that facilitates both steroidogenesis substrate availability and organelle product passage. Besides increased activity, the number and volume of these organelles are significantly higher in steroidogenic cells compared to other cell types (Lunstra et al. 1986; Mori and Christensen 1980). Many estrogen-mimicking chemicals targeting Leydig cell mitochondria disturb their ultrastructure, as we found in the present study, as well as mitochondrial steroidogenic enzymes and protein function, which results in serious Leydig cell and further testis dysfunction (Svechnikov et al. 2010). No data are available on PCBs effect on the morphology and activity of Leydig cell mitochondria. However, to date, the effect of other endocrine disrupters has been partially described. Histomorphological analysis of rat testes after exposure to di(n-butyl) phthalate revealed an increased Leydig cell number that showed swollen mitochondria (Ha et al. 2016). In animals, exposed to di(n-butyl) phthalate *in utero*, dose-dependent and age-related changes of Leydig cell mitochondria morphology, function and associated proteins were observed (Motohashi et al. 2016). Also, in tumors, Leydig cell organophosphate flame retardants affected mitochondrial activity, cell survival and superoxide production (Schang et al. 2016). Estrogen-like chemicals acted similarly in human liposarcoma cells (SW 872), e.g., mono-(2-ethylhexyl) phthalate affected mitochondrial translocated protein (TSPO), located in the mitochondrial membrane and coupled with steroidogenic acute regulatory protein (StAR) (Campioli et al. 2011). On the other hand, a mixture of 15 organochlorines did not affect mitochondrial activity in two mouse Leydig cell lines (MLTC-1 and MA-10) but targeted StAR cholesterol transport into the mitochondria and the conversion of cholesterol into pregnenolone inside the mitochondria by cytochrome P45011A- and NADPH-dependent adrenodoxin reductase (Enangue Njembele et al. 2014).

Zhong et al. (2015) demonstrated that Aroclor 1254 exposure suppressed cell viability and induced apoptosis in A549 cells (lung cell line). This was associated with reactive oxygen species overproduction and an elevated cellular Ca2+ level, which all resulted in mitochondrial membrane potential dysfunction.

Sex steroid hormones control mitochondrial function and dysregulation of steroid secretion and mitochondrial potential compromises cellular integrity and leads to a progressive decline in tissue function, e.g., during aging (Velarde 2014). Additional studies have shown a decrease in mitochondrial number and function correlated to age (Short et al. 2005). PCBs significantly increased Ca2+ concentration in Leydig cells but neither dose- nor type-specific effects were demonstrated. In chicken, after short-term (1000 mg/kg/7 days) and long-term (150 mg/kg/21 days) administration of d103, alterations in the Ca2+ level in blood were found (Piskac et al. 1990; Ruprich and Piskac 1990). In contrast, in piglets treated in the same scheme, no adverse effects on health condition, production parameters and concentration of blood molecules were revealed (Dvorák and Neumannová 1987). Thus, experimental design, including administration arrangements and studied parameters, performed analyses, type of chemicals as well as animal species, type of tissue or cell together with its age (e.g., immature or mature) and origin (e.g., primary or immortal) can significantly influence the obtained results. In bovine brain-derived endothelial cells, the effects of estradiol on intracellular Ca2+ homeostasis have been demonstrated (Suman et al. 2012). After estradiol exposure, the Ca2+ level significantly decreased, whereas the cytostolic and endoplasmic levels remained unchanged. A level of Ca2+ and Ca2+ fluxes, endocrine-disrupting chemicals effect on mitochondrial function and enzymes action all contribute to chemical-mediated developmental toxicity in animals and humans (Pretorius and Bornman 2005). In Leydig cells, inhibition of the electron transport chain increased intracellular Ca2+, which in turn resulted in an increase of testosterone production (Hales et al. 2005; Kumar et al. 1994; Sullivan and Cooke 1986; Tomic et al. 1995). Suppression of Ca2+ increased by hormonal and non-hormonal factors inhibits sex steroid synthesis. Estrogens have been suggested to inhibit the sodium-dependent efflux of Ca2+ ions from mitochondria (Santo-Domingo and Demaurex 2010). As a consequence of the increase in mitochondrial Ca2+ concentration, enhanced synthesis of reactive oxygen species was revealed. Estrogen-dependent tissues seem to be more susceptible to oxidative stress, resulting in DNA damage and, consequently, in higher mutation rates (Skibińska et al. 2016).

Sex steroids trigger a complex molecular mechanism controlling mitochondria function in endocrine steroidogenic cells, which involves crosstalk between the mitochondria, nucleus and plasma membrane as well as the cytoskeleton (Sewer and Li 2008). In the present study, elevated sex steroid levels in Leydig cells exposed to d103 and d106 were found, which indicates estrogenic properties of PCBs in Leydig cells. Similarly, Anbalagan et al. (2003) reported increased estradiol levels but no changes in testosterone levels, in rats treated for 30 days with Aroclor. In co-cultures of granulosa and theca cells, d103 first showed androgenic action but, after a longer exposure, it stimulated P450 aromatase activity (Gregoraszczuk et al. 2005). Moreover, Grabic et al. (2006) showed an increased secretion of estrogens by PCBs-exposed placental tissue. However, a decrease in the activity of microsomal enzyme C21 side-chain cleavage P450 by PCBs treatment of Leydig cells in vitro was demonstrated by Kovacević et al. (1995). Elumalai et al. (2009) and Murugesan et al. (2007) reported that Aroclor 1254 treatment significantly reduced the serum testosterone level, together with the expression of steroidogenic proteins and enzymes in exposed rats. Studies by Wojtowicz et al. (2001, 2005) showed an increase in testosterone secretion in granulosa cells and no changes in its level in co-cultures of granulosa and theca cells of exposed pigs. However, in long-lasting exposure (for 4 and 6 days), anti-estrogenic action of PCBs (126 and 153) with activation of AhR only in granulosa cells were noted. Gregoraszczuk et al. (2003) demonstrated an increased secretion of estradiol by granulosa cells derived from large pig follicles. Thus, it should be added to the above quoted information that various actions of PCBs on secretion of steroids strongly depend on the time of the exposure, cell type and cell development. In fact, according to Diamanti-Kandarakis et al. (2009), there are 209 different possible chlorine substitutions on the biphenyl backbone of PCBs, with the resulting PCB molecules having different structural, functional and toxicological properties. Taking into account both the latter and the previously provided information, our results showing dose- and type-dependent effects concomitantly with the common effect of delors on various aspects of Leydig cell function are not surprising. Understanding the diverse actions of delors on the function of Leydig cells, their organelle receptor proteins, secreted steroid hormones and production of other non-hormonal molecules needs further intensive studies and highly advanced research tools.

## Conclusion

The existence of possible relationships between ERRs expression and the ultrastructural and functional status of the mitochondria in Leydig cells is a crucial future research direction. Defining ERRs role in PCBs action, as well as in both endogenous and environmental estrogen signaling (together with discovering other molecules that activate ERRs) in the cells of the male reproductive system, will provide new insights into Leydig cell function in physiological and pathological states. This will be undeniably helpful for the identification of novel hormonal therapeutic strategies for infertility and cancer treatments.
